# Late subadult ontogeny and adult aging of the human thorax reveals divergent growth trajectories between sexes

**DOI:** 10.1038/s41598-020-67664-5

**Published:** 2020-07-01

**Authors:** Daniel García-Martínez, Markus Bastir, Chiara Villa, Francisco García-Río, Isabel Torres-Sánchez, Wolfgang Recheis, Alon Barash, Roman Hossein Khonsari, Paul O’Higgins, Marc R. Meyer, Yann Heuzé

**Affiliations:** 1 CNRS, MCC, PACEA, UMR5199, University of Bordeaux, Allée Geoffroy Saint Hilaire Bat. B8, CS 50023, 33615 Pessac Cedex, France; 2 Paleobiology Department, Museo Nacional de Ciencias Naturales (CSIC), José Gutiérrez Abascal 2, 28006 Madrid, Spain; 3 Centro de Estudios del Campo de Montiel (CECM), Plaza Mayor s/n, 13328 Almedina, Castilla-La Mancha Spain; 4 Centro Nacional Para El Estudio de La Evolución Humana (CENIEH), Paseo Sierra de Atapuerca 3, 09002 Burgos, Spain; 5 Laboratory of Advanced Imaging and 3D Modeling, Section of Forensic Pathology, Department of Forensic Medicine, University of Copenhagen, Frederik V’s vej 11, 2100 Copenhagen, Denmark; 6 Hospital La Paz Institute for Health Research (IdiPAZ), Paseo de la Castellana 261, 28046 Madrid, Spain; 7 Centro de Investigación Biomédica en Red en Enfermedades Respiratorias (CIBERES), Av. de Monforte de Lemos 5, 28029 Madrid, Spain; 8 Department of Radiology, Medizinische Universität Innsbruck, 6020 Innsbruck, Austria; 9 Faculty of Medicine Galilee, Bar Ilan University, Henrietta Szold, 1311502 Zefat, Israel; 10 Service de chirurgie maxillofaciale et chirurgie plastique, Hôpital Necker – Enfants Malades, Assistance Publique, Hôpitaux de Paris, Université de Paris, Paris, France; 11 Department of Archaeology and Hull York Medical School, The University of York, York, UK; 12 Department of Anthropology, Chaffey College, Rancho Cucamonga, CA 91737 USA

**Keywords:** Evolution, Anthropology, Evolutionary developmental biology, Palaeontology

## Abstract

Sexual dimorphism is an important feature of adult thorax morphology, but when and how sex-related differences in the ribcage arise during ontogeny is poorly known. Previous research proposed that sex-related size differences in the nasal region arise during puberty. Therefore, we explore whether ribcage sexual dimorphism also arises at that time and whether this sexual dimorphism is maintained until old age. We measured 526 (semi)landmarks on 80 CT-based human ribcage reconstructions, on individuals ranging from 7 to 65 year-old. The 3D coordinates were submitted to the Procrustes superimposition and analyzed. Our results show that the trajectories of thorax size and shape between sexes diverge at around 12 years of age, and continue slightly diverging until old age. The differential ontogenetic trends cause adult male ribcages to become deeper, shorter, and wider than female. Our results are consistent with the evidence from the cranial respiratory system, with the development of sexual dimorphism probably related to changes in body composition during puberty combined with changes in the reproductive system.

## Introduction

Over the last few decades, sexual dimorphism has been identified as an important factor underlaying the variation in form and function of the respiratory apparatus. Soft and hard tissues of the craniofacial respiratory system, the nasal cavity^1–10^, and the postcranial respiratory system, the ribcage^12–26^ have all been studied.

At the beginning of the XXI century, authors reported significant sexual dimorphism in the sagittal plane of the human skull for a European sample of around 100 individuals, with males having a relatively larger nasopharyngeal space than females, because of a larger piriform aperture, larger choanae and a more posteriorly inclined occipital clivus^2^. Other authors subsequently assessed sex-related differences in the nasal cavity of five different populations from different geographical regions, including cold-adapted populations^4^. Interestingly, they found that the sex-specific differences previously observed in the nasal cavity, such as the taller and larger piriform aperture and choanae observed in males compared to females^2^, were common (shared) features observed among human populations^4^. These differences were interpreted by the authors as an adaptation to greater oxygen and energetic demands in males compared to females, which is supported in a study of the fleshy nose that showed that “nose shape and volume, as well as other anthropometric measures, can be related to physiological measures of energetics”^3^. Therefore, because males have consistently larger absolute and relative lean body mass compared to females^27–29^, Bastir et al.^4^ associated the size and shape differences in the human nasal cavity with differences in body composition and energetic requirements. This is consistent with data on bioenergetics from other researchers who found that adult males in Western, Farming and Hunter-gatherer populations had increased total energetic expenditure (TEE) and physiological activity level (PAL) compared to females^30^.

Since the respiratory apparatus is an integrated system, sex-related differences observed in the cranial (upper) part are likely to be reflected in the thoracic (lower) part^31^. Accordingly, it has been demonstrated that sex-related differences can also be found in the adult ribcage size, shape, and function^12,13,37,38,14,17,18,32–36^. There is a consensus that the male ribcage is relatively wider and shorter than the female ribcage, with ribs that are more horizontally oriented in the sagittal plane^13,14,18^. Besides, different kinematic patterns have been observed between sexes^21,35^, with male ribcages and lungs expanding during breathing cycles more in the mediolateral direction (bucket-handle movements) than do female ribcages, which expand more in the superoinferior direction (pump-handle movements). The wider lower ribcage of males could reflect a more diaphragmatic breathing mode since the diaphragm is attached to this part of the thorax. This is supported by research on the thoracic-abdominal function where previous authors found that the force of abdominal movements was significantly stronger in males during deep breathing or vital capacity manoeuvers^37,38^.

While sex-related differences in the adult respiratory system concerning body composition and bioenergetics have been recognized and quantified, it is currently unknown when these differences arise during ontogeny. Do differences in the lower respiratory tract arise late in ontogeny, when differences in body composition become more apparent, or earlier? To the best of our knowledge, no prior study has addressed the ontogeny of sexual dimorphism of the skeletal part of the ribcage or the nasal cavity properly, but research on the ontogeny of the human fleshy nose and body composition can shed light on this issue. Previous studies^3,8,9^ reported that the divergence of external nasal morphology between sexes typically becomes apparent during puberty or early adolescence, i.e. around the age of 12, when differences in body composition and daily energetic expenditure (DEE) also become evident^28,29,39^. The ontogenetic scaling of external nasal size relative to body size is different for males and females because, as sitting height increases, males tend to exhibit a disproportionate increase in nasal height compared to females^8,9^. This trend was suggested to be associated with a greater increase in fat-free (lean) mass relative to the total mass in males relative to females from puberty onwards^29^. Plausibly, the greater percentage of fat-free body mass in males would require proportionately greater oxygen intake and thus greater flow, which might be achieved through larger nasal size^2–4,8,9^.

While knowledge of sexual dimorphism in the ontogeny of the nasal region is mostly restricted to the external fleshy nose, what we know about the sexual dimorphism of ribcage ontogeny is even more limited, likely due to the difficulty of obtaining ontogenetic series of CT scans^40,41^. Cynthia Beall^42–44^ studied anthropometric measurements such as thorax width and depth in living children from high altitudes and lowlands. Even though they recorded sex for the children, the focus of the study was to compare populations in the light of high altitude adaptation, only briefly tackling sexual dimorphism in ontogeny. From their data, it can be observed that boys had deeper and wider thoraes than girls throughout ontogeny^43^ in high altitude populations. Even though this information is important, it is not a proper ontogenetic study on sexual dimorphism and it has the limitation that linear measurements cannot accurately describe the entire 3D anatomy of the ribcage.

Other studies of respiratory system ontogeny have focused on calculating standard values for physiological variables such as the total lung capacity (TLC) in children^45–47^, rather than the form of the ribcage. Cook and Hamman^45^ found linear regressions of TLC on stature and showed that for the same height (range of 100–200 cm) and at every age, males had consistently larger TLC compared to females. This information was expanded on by other authors^47^, who found divergent trajectories for TLC between sexes from the age of around 13 onwards, but noted that this variable did not follow a linear trajectory throughout ontogeny; rather, it shows a rapid increase from around 7 years of age until around 18 years. It is striking to note the great similarity between the trend in TLC ontogeny from these authors^47^ (their Fig. 4; Supplementary Information Fig. S1 from this work), the ontogeny of nasal size^8^ (their Fig. 4; Supplementary Information Fig. S1 from this work) and the ontogeny of the fat-free mass index^29^ (their Fig. 1a; Supplementary Information Fig. S1 from this work). Finally, it is important to point out that the only study that focused on late subadult ontogeny combining physiological variables with thoracic linear measurements^46^, found divergent trajectories in ventilatory capacity (VC) between males and females from the age of 13 onwards, with males having progressively larger volumes than females throughout ontogeny. Furthermore, they found that male ribcages at the age of 13 were medio-laterally narrower and superoinferiorly shorter than the female ribcages, but this trend was inverted throughout late subadult ontogeny, with male ribcages becoming progressively wider and taller. However, it is important to note that the ribcage is a complex 3D structure and two linear measurements are insufficient to describe its morphology. For example, we could get very different results if we compare thorax width or depth at the level of rib 1, rib 7, or rib 12. What of those levels would give more reliable information about thorax size and/or shape? This prior work should be expanded by undertaking 3D analyses of ribcage form to better understand the ontogeny of sexual differences.

Other researchers have focused on the ontogeny of the ribcage from newborns to adults studying changes in the configurations of the upper and lower ribcage, but ignoring sex^40^. Specifically, they found that the lower ribcage undergoes a relative mediolateral narrowing during the transition from newborns to adults, whereas the upper part undergoes a relative expansion. They also challenged classical ideas that adult ribcage morphology was already established at the age of two since they found a curved ontogenetic trajectory that included modifications in the relative mediolateral and anteroposterior dimensions, the spine curvature or the sternum position in later ontogeny. However, they did not focus on sexual dimorphism in late subadult ontogeny, where important changes (based on information of the nasal part and physiological parameters) might be expected to occur. A study of thoracic biomechanics also found that ribcage three-dimensional morphology continues to change during adulthood, and proposed adult aging as an important factor in modifying the anteroposterior dimension of the ribcage and the rib orientation relative to the horizontal plane^48^. However, like Bastir et al.^40^, this study did not account for sexual dimorphism as a potential factor underlying variation in the adult aging process. Because energetic demands and body composition are different between sexes and between young and old^49–51^, and menopause affects females but not males^51,52^, it is possible that adult aging has different effects on ribcage form in each sex. Finally, other authors have suggested an important possible role of sex differences in reproductive anatomy and physiology in shaping thoracic dimorphism^12–14^.

Several lines of research suggest that we should expect sex-related differences in the ontogeny of ribcage form, but these are yet unknown in detail. This study aims to fill this gap in knowledge and explore (1) whether late subadult ribcage ontogeny parallels what is found in the nasal respiratory apparatus, and (2) if adult aging is associated with changes in ribcage form that differ between males and females.

## Results

### Changes in size: growth

Regression of the ribcage linear measurements on age shows divergent ontogenetic trajectories, with similar but not identical trends in each measurement (Supplementary Information Fig. S2). Anterior spine length (ASL) is the only measurement for which females show larger values than males. This appears to be below the age of 13 but is not marked, although we could not test for significance because of the limited sample size. From this age on, the ASL of females does not increase in size very much and soon ceases to grow, possibly even decreasing from the age of 40 onwards. In contrast, male spines undergo a more rapid increase in size during adolescence until they reach adulthood and do not decrease from the age of 40 onwards (Supplementary Information Fig. S2).

Both males and females exhibit similar absolute values of thorax width (TXW) at the age of seven, but the trajectories immediately begin to diverge, with males showing a more rapid increase in TXW with age than females. Also, from around the age of 40, female TXW begins to decrease, whereas in males it continues to increase slightly with age (Supplementary Information Fig. S2). Finally, in thorax depth (TXD), from 7 to 13 years of age, both sexes follow parallel trajectories that diverge as males grow faster to achieve adulthood. Afterward, the difference in TXD remains relatively constant until old adulthood. It is interesting to note that this is the only variable in which females do not show a decrease with adult aging, even possibly increasing from 40 onwards, similar to the condition in males (Supplementary Information Fig. S3).

Centroid sizes (Supplementary Information Fig. S3) show similar trends to those of linear measurements: CS is very similar at the age of seven, but after that, the CS trajectories diverge because of a more rapid increase in CS in males until adulthood is attained (around the age of 20). After this point, size differences remain approximately constant until the age of 40, and then males increase and females decrease in size with adult aging (Supplementary Information Fig. S3). That males manifest greater growth velocities than females between the ages of 7 and 21 is evident from a plot of percentage size increase per year (Supplementary Information Fig. S4); male growth is much larger than in females when young but these become more equal as adulthood is attained (Supplementary Information Fig. S4).

When CS is standardized by stature, we observe that subadult male ribcages are similar or even disproportionately small compared to the female ones, but this trend is inverted over the course of ontogeny, so male ribcages in adults are disproportionately large for their stature compared to the female ribcages (Supplementary Information Fig. S5).

### Changes in form: development

**Figure 1:**
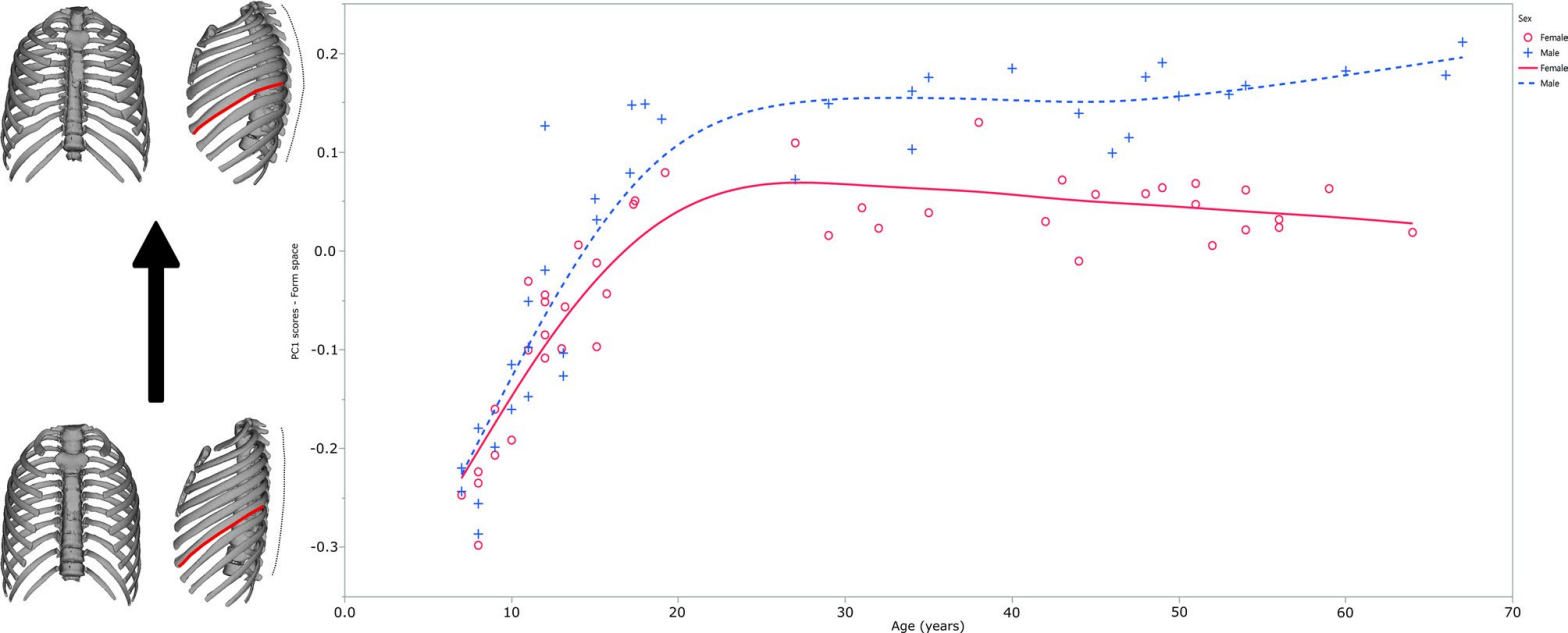
Ontogenetic variation of Form space PC1 (70% of the variability of the sample with age) showing divergent trajectories between males (blue; dashed line) and females (red; simple line). To the left, 3D morphological variation in the ribcage related to PC1 form space scores is visualized (bottom – negative, top – positive). To better visualize the variation in lateral view, the black dashed line shows the spine profile and the simple red line shows rib twisting at the 7 rib.

PC1 of form space, accounting for 70% of the total variance of the sample, relates to variations in size and shape with age (ontogenetic allometry), including variation in relative spine curvature, rib torsion, thorax width, and depth. When plotted against age, differences between males and females are most marked in late subadult ontogeny (Fig. 1; Supplementary Information Figs. S6-7).

**Figure 2:**
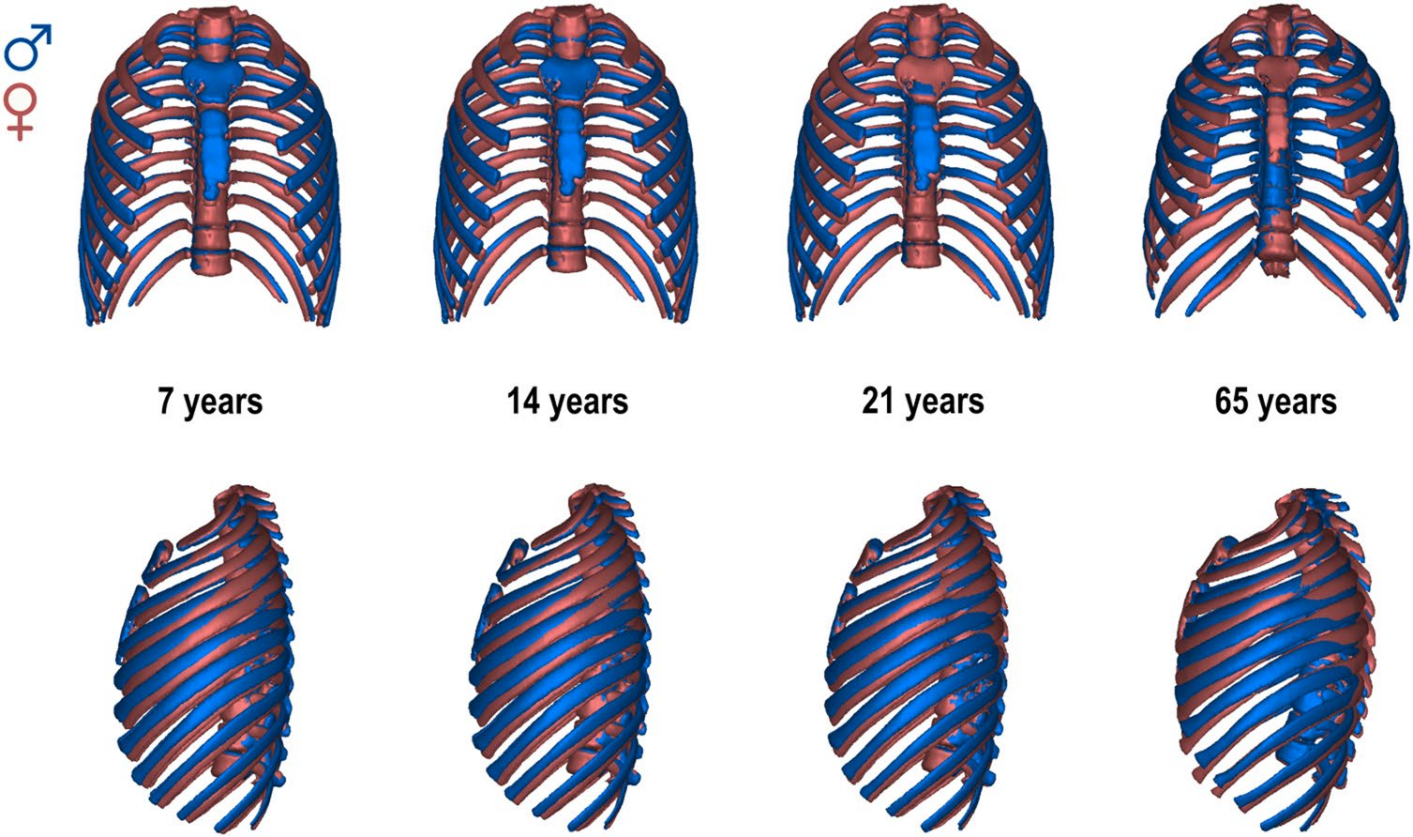
Morphological differences between males (blue) and females (red) at the ages of 7, 14, 21, and 65 year-old. The ribcages are shown in Procrustes superimposition, so only shape differences can be observed.

**Figure 3:**
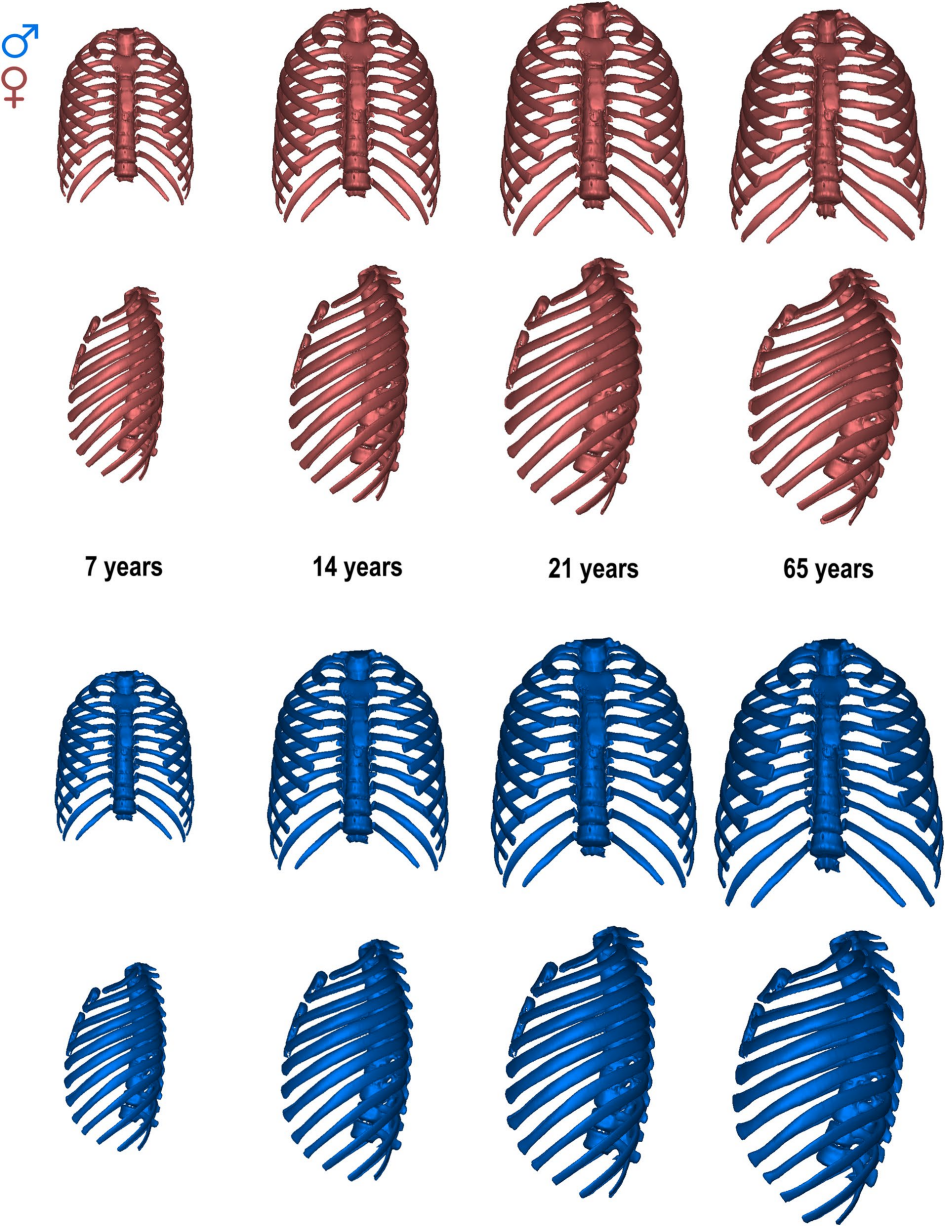
Morphological differences between males (blue) and females (red) at the ages of 7, 14, 21, and 65 year-old. The ribcages are shown on a real scale, so size and shape differences can be observed.

By the age of seven, both males and females have ribcages that are anteroposteriorly flat and medio-laterally narrow (Figs. 1, 2, 3). They also exhibit less curvature of the thoracic spine than older adults and less twisting of ribs compared to them, features that can be observed in lateral view (Fig. 1). However, as ribcages grow, their morphology is modified differently in each sex: male ribcages become relatively wider in the lower part and the thoracic spine of males becomes relatively shorter throughout ontogeny (Fig. 1). Since PC1 does not fully account for ontogenetic shape changes, more detailed information can be obtained when we standardize the morphology of the ribcage in both sexes, using their corresponding trajectories of full shape on age (Supplementary Information Figs. S6-7). Ribcages standardized to the ages of 7, 14, 21 and 65 years allow us to explore detailed changes related to late subadult ontogeny and adult aging (Figs. 2, 3).

There are features of sexual dimorphism that are present regardless of age, both in late subadult ontogeny and old age, such as the relative widening of the ribcage in males coupled with the relative shortening of their thoracic spines (Figs. 2, 3). However, other features are not constant throughout ontogeny. This is the case for the position of the sternum, which is more anteriorly positioned in males than in females in juveniles and adolescents but not in adults, or the thoracic spine, which is equally curved in males and females in juveniles and adolescents but relatively more curved in females than in males in older individuals (Figs. 2, 3). It is important to state that PC1 accounts for variation in the spine, but not all the variation is included in that PC. This might be the reason that females, with more negative values of PC1, have a more curved spine after the by-age standardizations than males. Besides, the ribcage is relatively deeper in males than in females at every stage except for the older individuals, in which female ribcages are relatively deeper than male ones (Figs. 2, 3). This ontogenetic trend can be linked to the fact that thorax depth is the only female linear measurement that does not decrease with adult aging (Supplementary Information Fig. S1). These differences in ontogeny of the ribcages of males and females are reflected in divergent ontogenetic trajectories between sexes that affect both young and older individuals.

## Discussion

### Sexual dimorphism in modern human late subadult ontogeny and its potential physiological importance

How the shapes and sizes of the different parts of the respiratory system, vary with ventilatory function, are key to understand the evolution and development of human breathing. Respiration rate (fR) and differences in breathing kinematics^21,35,53^ will also play a key role in increasing ventilation in response to energetic needs. It has been hypothesized that late subadult ontogeny and sexual dimorphism are crucial to understanding the development of the respiratory system, due to the association between respiratory and energetic demands and body composition^28,29^. Therefore, to fulfill those demands, respiratory apparatus grows both in its craniofacial (nasal cavity and fleshy nose) and post-cranial (ribcage) parts. Most studies agree that sex differences in the respiratory system become more apparent around the age of 12–13. Specifically, some previous researchers^3,8,9^ found that divergence in external nasal morphology arises around early adolescence (12 years), which corresponds with the findings of Stocks & Quanjer^47^ that divergent trajectories in total lung capacity between boys and girls arise around the age of 13. Therefore, for these two parts of the respiratory system, adolescence marks a change in the degree of dimorphism.

We find that the growth and development of the ribcage, as in external nose size and total lung capacity, show a marked increase in dimorphism from 12 to 13 years of age onwards, which is attributable to male acceleration of change during puberty and early adolescence compared to females (Supplementary Information Figs. S1-S3). This rapid increase is particularly evident in thoracic spine length, the only variable that is smaller in males than in females at the age of 7 but which becomes much larger in males throughout ontogeny (Fig. 1). This length increase could be related to somatic growth reflected in anthropometric variables such as trunk length, leg length, and sitting height, confirming the differential development between males and females that we observed in the ribcage^54–57^. This length increase during the so-called growth spurt could also be related to the rapid TLC increase previously detected^47^.

Regarding sex-related ontogenetic changes in shape, DeGroodt et al.^46^ agreed that between 7 and 12 years old, male ribcages are absolutely and relatively shorter than female. However, they found that at 13 years old, male ribcages are medio-laterally narrower and shorter than female, which does not correspond with our results. This discrepancy might be a function of methodological differences since they quantified external measurements in living subjects whereas we quantify skeletal dimensions from CT scans. Bastir et al.^40^ found that thoracic ontogeny followed a curved trajectory that gives rise to an increase in the mediolateral thoracic dimension and increased spine curvature, among other features. However, they did not account for sex in their study. Here we add that sex-specific differences underlie aspects of variation in late subadult ontogeny. Our results show that the mediolateral expansion of the ribcage is more pronounced in males than in females at every age from seven onwards. Our findings also indicate that the thoracic spine becomes more curved throughout ontogeny (from 7 until old age), but females develop greater spinal curvature than males. The greater thoracic vertebral kyphosis seen in females may functionally compensate for their greater lumbar lordosis since it helps to bring the center of mass of the upper body back over the hip joints^58–60^. In this study, we also find that spine length is relatively shorter in males than in females and that thoracic depth is relatively larger in males than females at every ontogenetic period except in old age, when female thoracic depth is greater than that of males.

Several studies also noticed a differential increase in DEE around adolescence^28,39^, which could be linked to the changes in size and shape observed in the ribcage (our results) and the nasal cavity^8,9^. For example, Bitar et al.^28^ found clear gender differences in body composition and DEE between 10.4 and 12.8 years. Importantly, even though their sample was limited, they noticed striking differences related to Tanner’s stage^61^ in boys at the onset of puberty, suggesting that sex hormones differences may play an important role in changes in DEE^28^. This is consistent with further research^39^ that found that DEE increased significantly with age in boys, but not in girls. Specifically, DEE of male adolescents increased significantly between 12.6 and 15 years, whereas DEE of female adolescents reached a plateau at 12.6 years. They linked this difference to body composition since they found no significant differences in these variables between boys and girls at around 13 years, but these differences became significant at the age of 15. They pointed out that, apart from these differences in body composition, the physical activity level (PAL) in young males and females was crucial for understanding the reported differences in DEE^39^. This was in line with previous research on DEE and PAL in boys and girls, supporting also a larger degree of PAL in boys compared to girls^62–67^. Unfortunately, the nature of our data does not allow us to specifically test the correlation between thorax size/shape and DEE or PAL, but this relationship could be inferred from the above-mentioned data.

Finally, it is important to mention that our results on potential respiratory differences between males and females are, partially challenged by recently published work^68^ on sex-specific differences in respiratory function. Although significant reductions in peripheral capillary oxygen saturation (an estimate in the amount of oxygen in the blood; SpO2) during walking were observed in environments with normal levels of oxygen (21%) and reduced levels of oxygen (13%), sex differences in SpO2 changes were not found. The reduction in SpO2 during exercise (exercise-induced arterial hypoxemia; EIAH) has been attributed to the ventilatory limits of the respiratory system. Horiuchi et al.’s^68^ findings that there are no sex-specific differences in SpO2 go against expectations that the smaller respiratory anatomy found in females and greater mechanical ventilatory constraints imposed on females predict more severe or frequent reductions in SpO2 (i.e. EIAH) in women than in men. Furthermore, as noted by Horiuchi et al., other studies have also shown no differences in EIAH between sexes in response to exercise^69^. As such, the role of mechanical constraints in EIAH should be reassessed. If Horiuchi et al. are correct, the mechanical and morphological differences between male and female modern humans may not be a result of differences in energetics or differing amounts of necessary ventilation because differences in sex-specific thoracic shape may not result in any difference in blood oxygen concentration.

In this regard, it is also important to discuss the potential importance of the reproductive system in shaping lower thorax morphology. Bellemare et al.^13^, on the sexual-dimorphism of the thoracic dimensions, proposed a disproportionate growth of the ribcage compared to the growth of the lungs, which would be consistent with the ability to accommodate abdominal distention during pregnancy in females. In their subsequent study^14^, on the sex-differences of the human ribs, proposed that the ribcage accommodates volume displacement not only during breathing but also during pregnancy in women. They found relatively longer ribs in females compared to males, which would allow a relatively greater rib cage expansion, well suited to accommodate the large abdominal distension that occurs during pregnancy. It is important to note that during late-stage pregnancy the fetus, placenta, and amniotic fluid limit the inferior displacement of the diaphragm, necessitating a greater emphasis on superoinferior (“pump handle”) movement of the upper thorax to ventilate the lungs. Selection may have favored a greater emphasis on the pump-handle, upper rib movement in the ventilatory kinematics of females, such that it is expressed (both in ribcage morphology and kinematics) in non-gravid^35,70,71^ as well as gravid females^70,71^. At puberty, elevated levels of osteogenic sex steroids begin to create sex differences in pelvic shape^72–75^, which are almost certainly related to reproduction and not to sex differences in oxygen demands. These same hormones may initiate the divergence in thoracic morphology between males and females and may be responsible for their divergence throughout life. The two hypotheses (energetic vs. reproductive) are not mutually exclusive.

Finally, Gayzik et al.^48^ found that anteroposterior expansion of the ribcage coupled with rib lowering occurs from 20 to 80 years of age. In our study, we also note sex differences and divergence of ontogenetic trajectories (Fig. 1 and Supplementary Information Figs. S1-S3). Thus, from 21 to 65 year-old, the anteroposterior expansion of the ribcage and rib lowering are more pronounced in females. Also, females develop a more curved spine than males as they age. Since hormone-mediated factors such as menopause only affect females^51,52^ and ribcage morphology and the spine is susceptible to osteoporosis, which is more prevalent in females^76–79^, those factors could likely impact female thorax size and shape. Besides, because energetic demands and body composition also differ between sexes at old ages^49,50^, it is possible that these differences could drive, at least partially, late-developing dimorphic features. It is important to state here that only one 65 year-old female individual is included, so future studies should include more individuals of approximately this age to confirm our results.

### Significance of our results for the study of human evolution

Through the course of human evolution, the function of the thorax has changed due to selection for increased ventilation in response to endurance running and increased energetic requirements, so energetics and ventilatory needs have altered the function of the thorax in humans and other hominins^20,53,80,81^. The fact that the late ontogeny of the ribcage is impacted by sexual dimorphism is important to take into account when studying the fossil record.

As far as we know, *Australopithecus* has been hypothesized as a dimorphic lineage, information that is partially provided by its thoracic material. For example, sexual dimorphism is used to explain why the thoracic material of the *A. afarensis* A.L.288-1 specimen, hypothesized as female^82^, is smaller than the thoracic material of the *A. afarensis* KSD-VP-1/1, hypothesized as a male^83^. Besides, sexual dimorphism is used in *A. sediba* to explain why the ribs and vertebrae of the MH1 specimen, a supposed juvenile male, is about the same size as the thoracic skeleton of MH2, a supposed adult female^84–86^. In this specific case, it is even more important to understand the ontogeny of sexual dimorphism in the ribcage, because the interpretation of what the hypothetical adult thorax of MH1 would have looked like as an adult is directly impacted by its sex. If we assume that sexual dimorphism observed in modern humans is the same as in *A. sediba*, we would not expect different interpretations depending on its sex. However, if we want to carry out developmental simulations of its thoracic material, sexual dimorphism should be accounted for.

This same logic is applied to other juvenile specimens from the hominin fossil record, such as the *H. erectus* from Nariokotome KNM-WT 15000^87,88^. This individual preserves enough thoracic material to make interpretations about its thorax, currently proposed as modern human-like^89^. However, for the interpretation of the thoracic morphology of this individual, it is important to take into consideration its subadult ontogenetic status^90–93^. If sexual dimorphism also affected the ribcage of *H. erectus*, very likely if it influenced *Australopithecus* and modern humans, and KNM-WT 15,000 had a skeletal age around 12–15 years^94^, likely, sexual dimorphism was already configuring its ribcage morphology. This is because in modern humans at this skeletal age, sexual dimorphism is important to take into consideration according to our results.

Understanding how sexual dimorphism impacts late ontogeny of the thorax, could be also important for the study of the Neanderthal ribcage. Sexual dimorphism is also well documented in Neanderthals^95–102^ and it can be noticed in costal size^32,103–105^. Unfortunately, even though the Neanderthal adult thoracic record is fairly large^104–109^, only the juveniles from El Sidrón site preserve thoracic material^110^. Those individuals have been hypothesized as around 7–9 years old, so if they followed a modern human pattern of thoracic dimorphism, their ribcage morphology was only slightly impacted by sexual dimorphism.

Finally, it is important to note that the populations surveyed here for the ribcage or by other authors for TEE and PAL are all subsamples of post-industrial Western populations, which might not fully represent what can be found in more traditional populations/lifestyles that were present over the vast majority of human evolution. Future studies should account for this issue.

### Conclusions

Our results point to a close relationship between the ontogeny of craniofacial and postcranial respiratory systems, as previously proposed^31^. Although similarities in sexual dimorphism in ontogenetic trends of TLC^47^ (their Fig. 4; Supplementary Information Fig. S1 from this work), nasal size^8^ (their Fig. 3; Supplementary Information Fig. S1 from this work) and fat-free mass index^29^ (their Fig. 1a; Supplementary Information Fig. S1 from this work) have already been noted in prior work, here we similarly find the expression of dimorphism in the ontogeny of the ribcage (Fig. 1 and Supplementary Information Figs. S1-S3). This finding highlights the importance of sexual dimorphism for development, as well as the close link between the craniofacial and the post-cranial respiratory system and body composition in terms of lean (fat-free)/fat mass and bioenergetics. It is important to state that we did not include FFM, a variable to be studied in our individuals because our aim is to study growth and development in the ribcage. The link between ribcage, nasal cavity, and FFM ontogeny is inferred according to other researchers´ results, and future studies should address ribcage 3D ontogeny and its correlation with variables such as fat-free mass and daily energetic expenditure (DEE). Finally, we also add the finding that females and males develop differently shaped ribcages and spines in adulthood.

## Material and methods

Tomography images (CT scans) of the ribcage of 81 Caucasian individuals balanced for sex were collected. The age groups were established^111^ and included 20 juveniles, 22 adolescents, and 39 adults (including elders), thus allowing us to assess late subadult ontogeny and adult aging. It is important to mention that we use the term “late subadult ontogeny” as used before^112^ -so from M1 (6–7 years) to M3 eruption (17–21 years)-, and the term “adult aging” as the gradual process of change from M3 eruption onwards. It is important to note that the term “aging” refers to the continuum that starts at birth and ends at death^113^, and should be differentitated from the term “senescence”, defined as the process of postreproductive aging (generally manifested as a decline in vitality and function)^113^, which is not the focus of this study. Sex was balanced in the sample and a visual inspection did not reveal signs of skeletal morphological alterations due to pathologies. Detailed information (age, sex, ontogenetic status, and scanning institution) about sample composition can be found in Supplementary Information Table S1. The juvenile and adolescent individuals were scanned at the Medizinischen Universität of Innsbruck (Innsbruck, Austria), the Department of Forensic Medicine of the University of Copenhagen (Copenhagen, Denmark) and the Hôpital Necker – Enfants Malades (Paris, France). Most of the adult individuals were scanned at the University Hospital of La Paz (Madrid, Spain), except for four individuals who were scanned at the Mount Carmel Hospital (Haifa, Israel), five individuals at Medizinischen Universität of Innsbruck (Innsbruck, Austria) and one individual at the New York City Hospital (NYC, U.S.). Approval to use these pre-existing CT-scans for the present study was granted by the different institutions’ IRB and all CT-data were anonymized to comply with the Helsinki declaration^114^.

Ribcages were segmented through a semi-automatic protocol for DICOM images using the open-source 3D Slicer software (https://www.slicer.org/) and subsequently reconstructed as 3D models. These 3D models were imported into Viewbox 4.0 software (www.dhal.com)^115^ for (semi-)landmarking using previously published protocols for ribs 1–12 and the thoracic spine^35^. The thorax morphology was quantified using 526 tridimensional landmarks and sliding semilandmarks: 7 landmarks and 13 sliding curve semilandmarks were placed on each rib 1–10, and 5 landmarks, and 13 sliding curve semilandmarks on each rib 11 and 12. Also, four landmarks were placed on the mid-sagittal plane of each thoracic vertebra and two additional landmarks were placed on the mid-sagittal plane of the sternum. The detailed information can be found in elsewhere^35^. The semilandmarks were slid along their corresponding curves concerning the fixed landmarks to minimize bending energy (BE), first using a random specimen of the sample as a reference for sliding and a second time using the consensus of the sample as a reference^116,117^.

Ribcage size was quantified following a double approach. First, we measured linear distances: anterior spine length (ASL; distance between the anterosuperior-most point of the T1 and the anteroinferior-most point of T12), thorax width (TXW; quantified as the distance between the semilandmark 10, located in the midshaft of the rib 7 of each side) and thorax depth (TXD; distance from the dorsal-most tip of the spinous process of T5 to the distal end of the rib 5, averaging both sides). Second, we compared centroid sizes (CS) to assess the general difference in size. CS is defined as the square root of the sum of squared distances of the set of landmarks from their centroid^118^, and so considers all the landmarks. To explore differences in the ontogenetic trajectories of thoracic size between sexes, we plotted ASL, TXW, TXD, and CS against age and quantified the trajectories using locally estimated scatterplot smoothing (loess) regressions. Besides, the thoracic growth rate (the size increase per year) was compared between sexes between the ages of seven (M1 erupted) and 21 (M3 erupted) (Supplementary Information Fig. S4). CS was also standardized by stature, which was measured using anthropometrical standard techniques in most of our sample and was estimated using a regression of T11 vertebral height and stature in the rest of the individuals. All this information can be found in Supplementary Information Fig. S8 and the Supplementary Information Table S1.

Shape data were obtained by generalized Procrustes analysis (GPA) of the entire landmarks and semilandmarks configuration^118^. Differences in ontogenetic trajectories were explored in the form space principal component analysis (form space PCA)^119^, which includes the natural logarithm of centroid size (lnCS), and therefore is a joint analysis of size and shape variation. Specifically, we studied the distribution of the form space PC1 scores on age to test for different ontogenetic trajectories in both late subadult ontogeny and old age. The morphological variation accounted for by PC1, which is driven by the size and thus, ontogeny, was visualized using the EVAN Toolbox (version 1.71; https://www.evan-society.org/). Finally, to study the detailed sex-specific allometric differences at different ontogenetic stages, we standardized the thoracic morphology of each sex by multivariate regression analysis to the ages of seven (juvenile), 14 (adolescent), 21 (adult) and 65 year-old (old adult) in the EVAN Toolbox. Before that, since the ontogenetic trajectory of both sexes studied here was curved, we split the curved trajectories into two linear subtrajectories, from 7 to 20 years of age and 21 to 65 year-old. The decomposition of a curved trajectory into linear ones, an approach followed by other authors^120^, allowed us to use linear regressions for calculating ribcage shape at different ages (Supplementary Information Figs. S6-S7).

## Supplementary information

Supplementary information

Supplementary information

## Data Availability

The data will be available upon request to the authors.
