# Supplementary material for: Late subadult ontogeny and adult aging of the human thorax reveals divergent growth trajectories between sexes
Source: Sci Rep. 2020 Jul 1;10:10737. doi: 10.1038/s41598-020-67664-5 (PMC7329879; doi:10.1038/s41598-020-67664-5)
Supplement: Supplementary file 1 — Supplementary information [file 41598_2020_67664_MOESM1_ESM.docx]

**Supplementary Online Information**

**Late subadult ontogeny and aging of the human thorax reveals divergent growth trajectories between sexes**

**Authors:** Daniel García-Martínez^1,2,3,4^, Markus Bastir^2^, Chiara Villa^5^, Francisco García-Río^6,7^, Isabel Torres-Sánchez^6^, Wolfgang Recheis^8^, Alon Barash^9^, Roman Hossein Khonsari^10^, Paul O’Higgins^11^, Marc R. Meyer^12^, Yann Heuzé^1^

^1^ University of Bordeaux, CNRS, MCC, PACEA, UMR5199, Allée Geoffroy Saint Hilaire, CS 50023, 33615 (Pessac, France)

^2^ Paleobiology Department, Museo Nacional de Ciencias Naturales (CSIC), José Gutiérrez Abascal 2, 28006 (Madrid, Spain)

^3^ Centro de Estudios del Campo de Montiel (CECM), Plaza Mayor s/n, 13328 (Almedina, Castilla-La Mancha, Spain)

^4^ Centro Nacional para el Estudio de la Evolución Humana (CENIEH), Paseo Sierra de Atapuerca 3, 09002 (Burgos, Spain)

^5^ Laboratory of Advanced Imaging and 3D modeling, Section of Forensic Pathology, Department of Forensic Medicine, University of Copenhagen, Frederik V's vej 11, DK-2100 (Copenhagen, Denmark)

^6^Hospital La Paz Institute for Health Research (IdiPAZ). Paseo de la Castellana 261, 28046 (Madrid, Spain)

^7^ Centro de Investigación Biomédica en Red en Enfermedades Respiratorias (CIBERES), Av. de Monforte de Lemos 5, 28029 (Madrid, Spain)

^8^ Department of Radiology, Medizinische Universität Innsbruck, 6020 (Innsbruck, Austria)

^9^ Faculty of Medicine Galilee, Bar Ilan University, Henrietta Szold, 1311502 (Zefat, Israel)

^10^ Hôpital Necker – Enfants Malades, Service de chirurgie maxillo-faciale et plastique, Université Sorbonne Paris Cité, Université Paris Descartes (Paris, France)

^11^ Department of Archaeology and Hull York Medical School, the University of York (York, United Kingdom)

^12^ Department of Anthropology, Chaffey College, Rancho Cucamonga, CA, 91737, USA


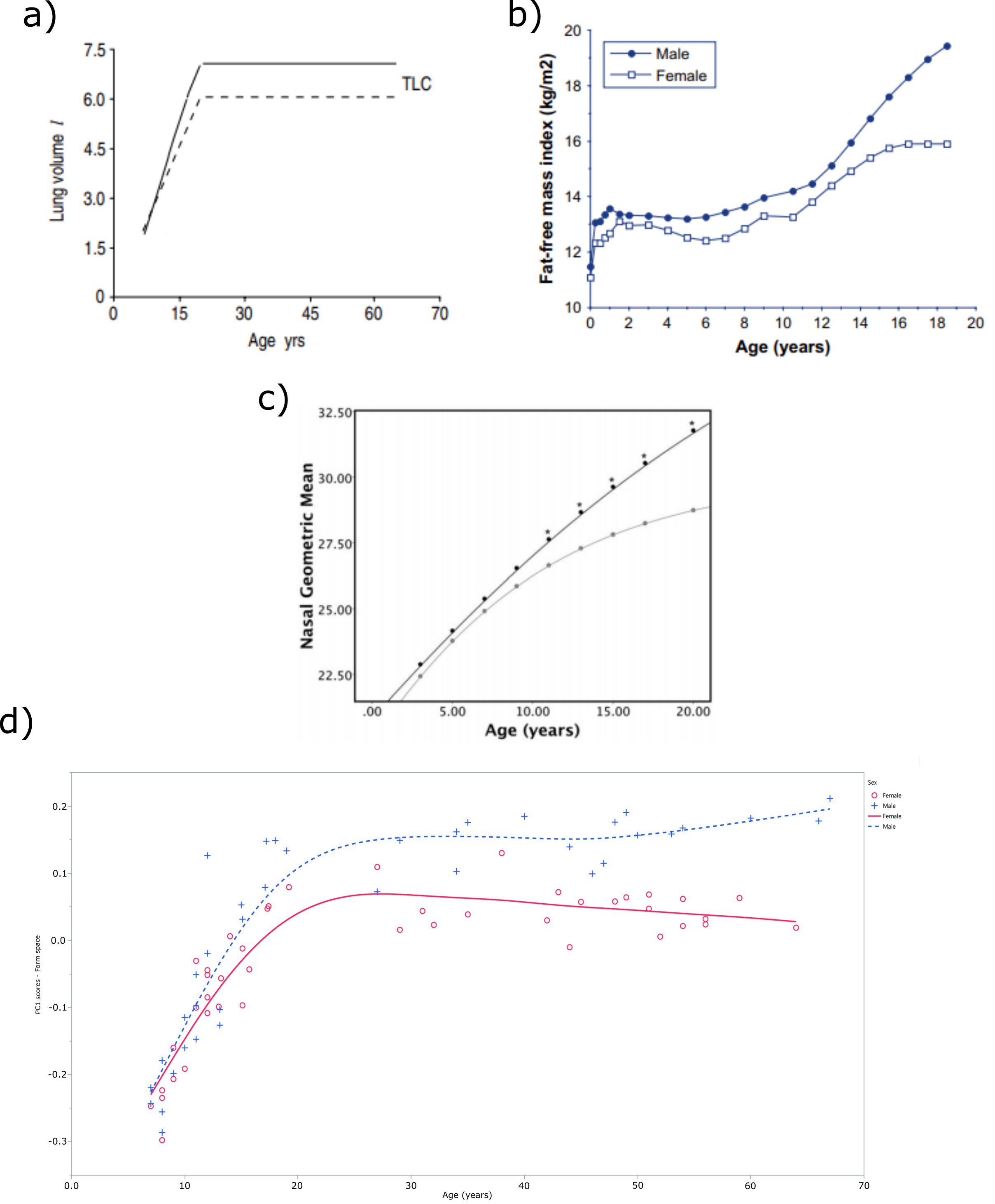


**Figure S1:** composite figure depicting similar dimorphic trends in the ontogeny of the a) total lung capacity, b) fat-free mass index, c) nasal size, and d) thorax form. Composite figure modified from the previous authors^45,9,29^ and combined with our data.


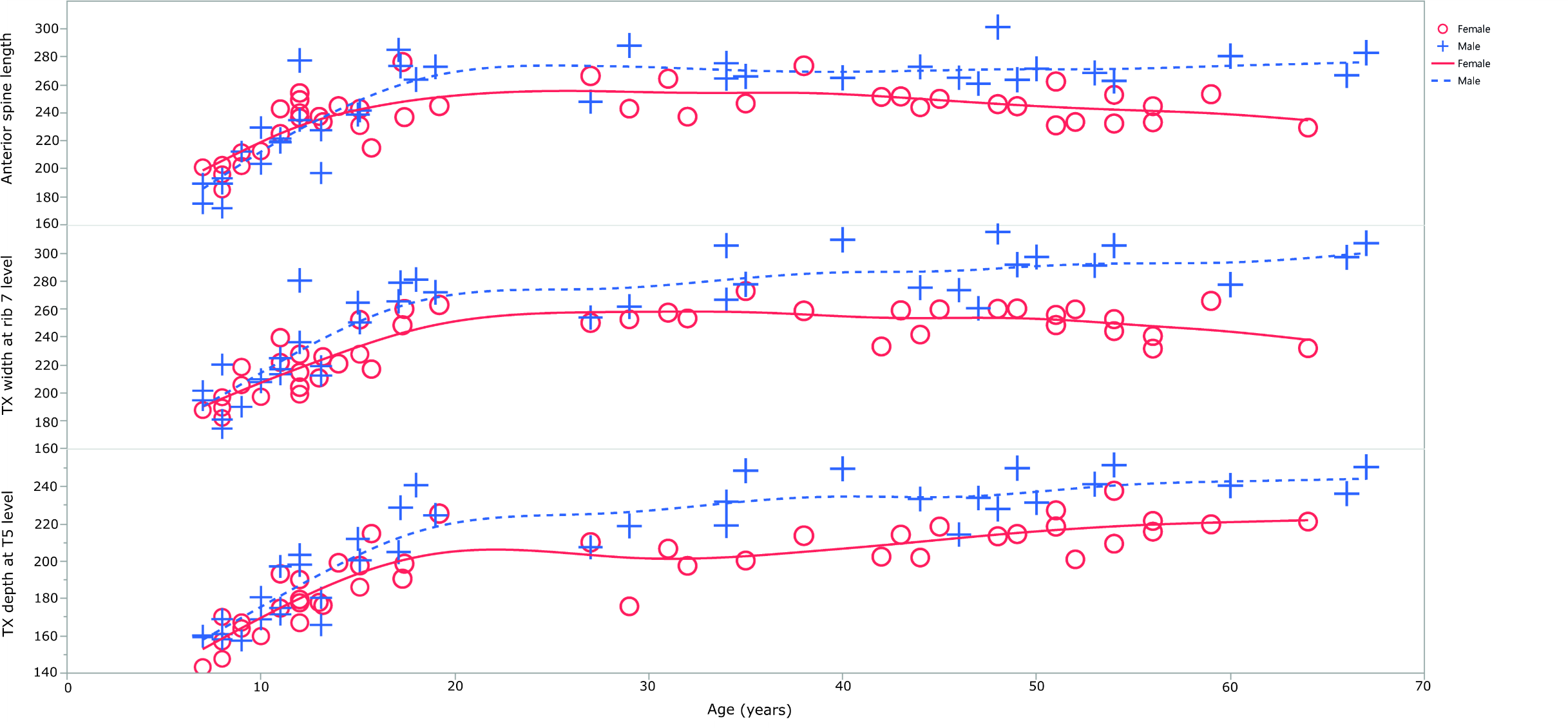


**Figure S2:** Variation of linear measurements (mm.) anterior spine length (top), thorax width (middle) and thorax depth (bottom) with age in males (blue; simple line) and females (red; dashed line). Divergent ontogenetic trajectories can be found in all of them.

**
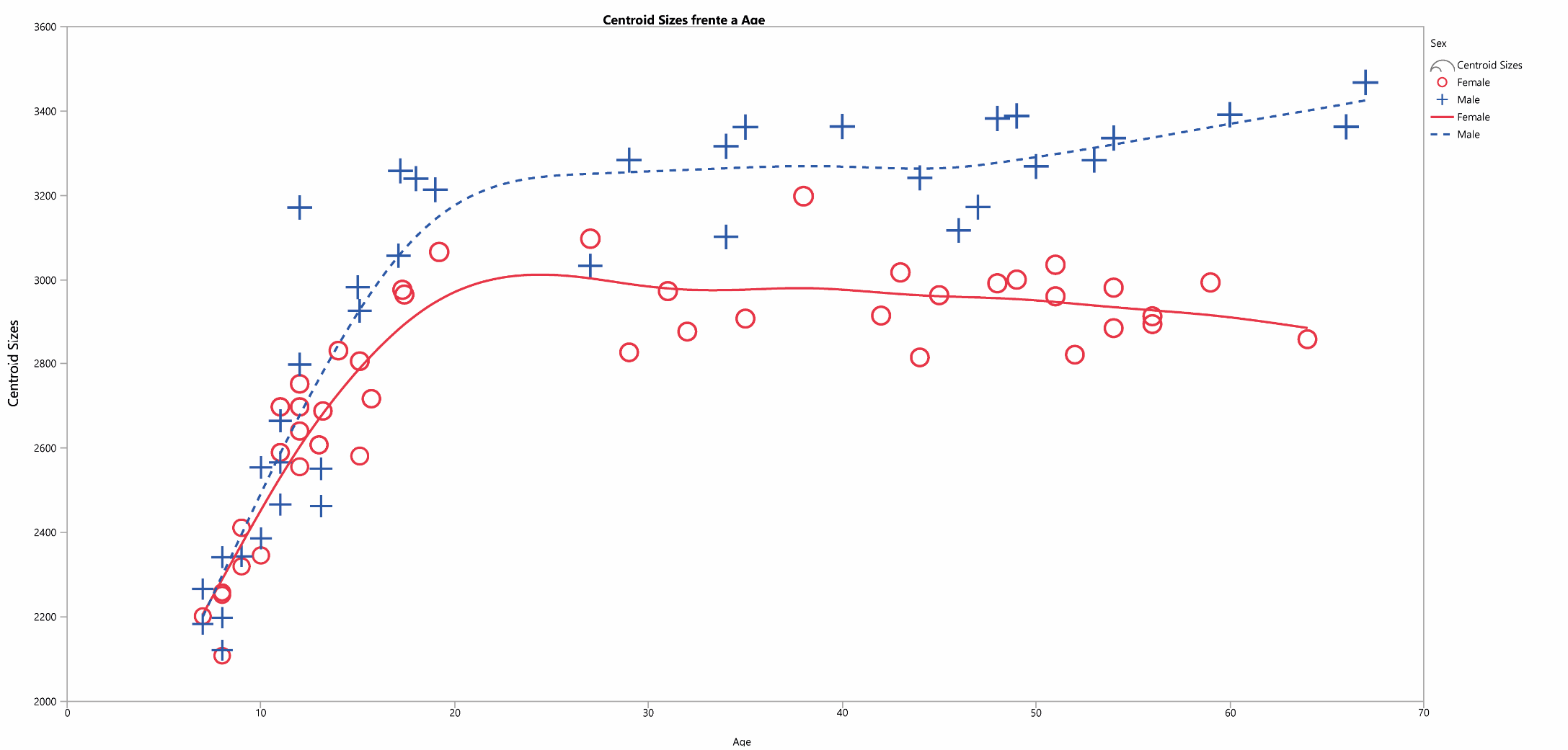
**

**Figure S3:** ontogenetic variation of CS with age, observing divergent trajectories between males (blue; simple line) and females (red; dashed line).

**
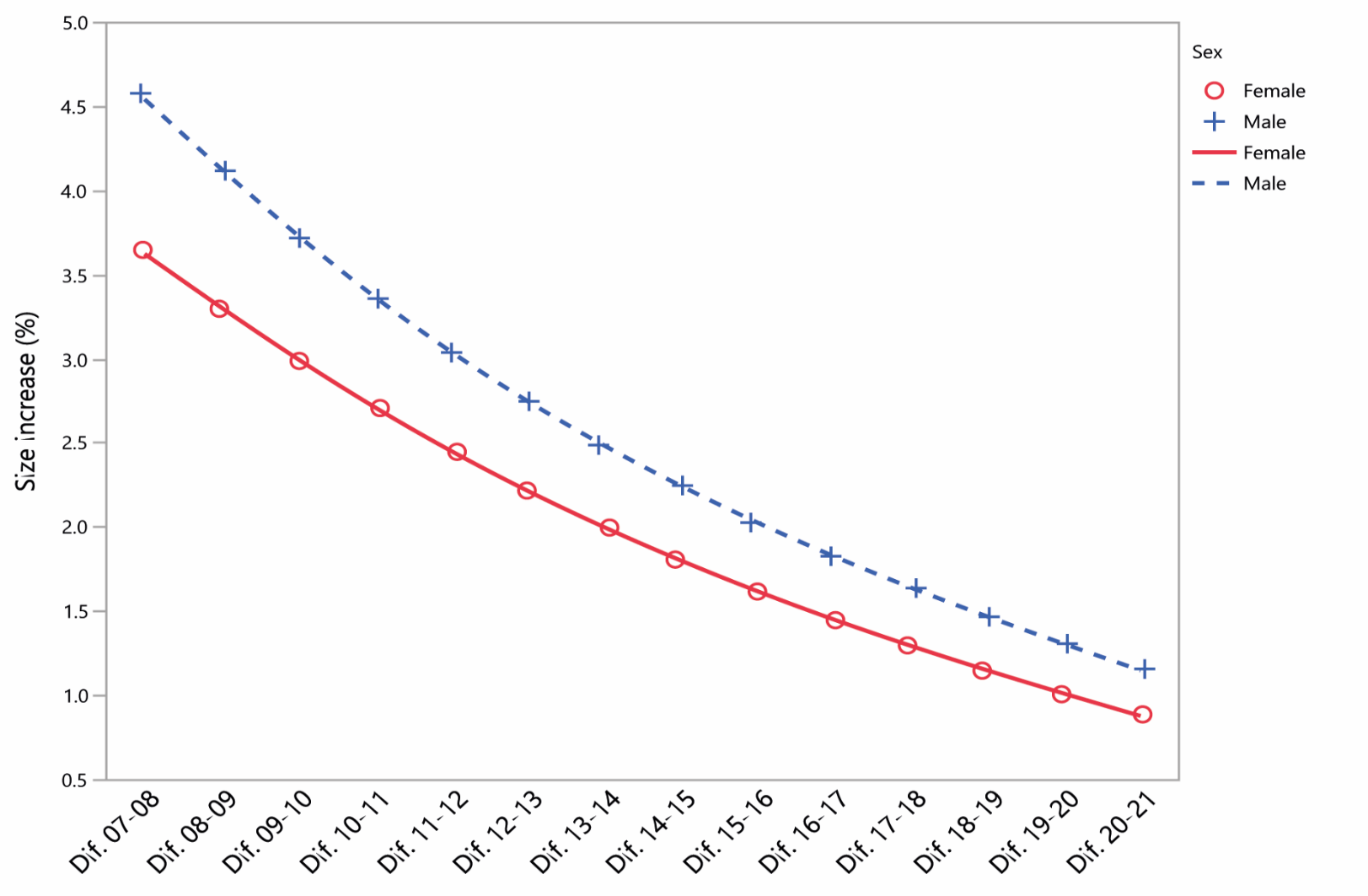
**

**Figure S4:** ontogenetic CS increase (%) in males (blue; simple line) and females (red; dashed line), observing a more rapid ontogenetic size increase in males than in females, which is more evident at the initial part

**Figure S5:** polynomial regressions of CS/stature ratio (y-axis) on age (x-axis), showing males (blue) and females (red) for the entire part of the ontogeny studied here.


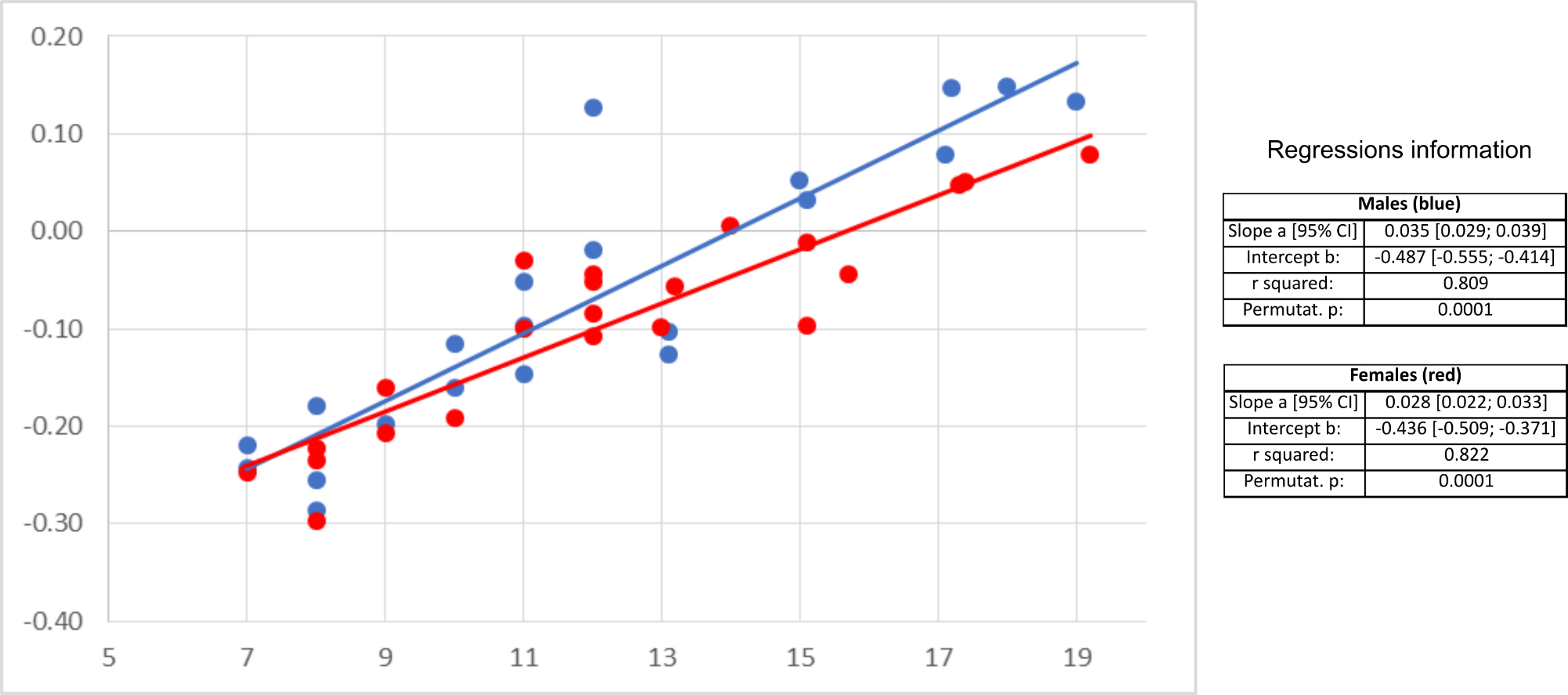


**Figure S6:** First part of human thorax late ontogeny. Depicted as linear regressions of shape scores (y-axis) on age (x-axis), showing males (blue) and females (red) subadults (from 7 to 20-year-old). Regressions information is shown in the right part.


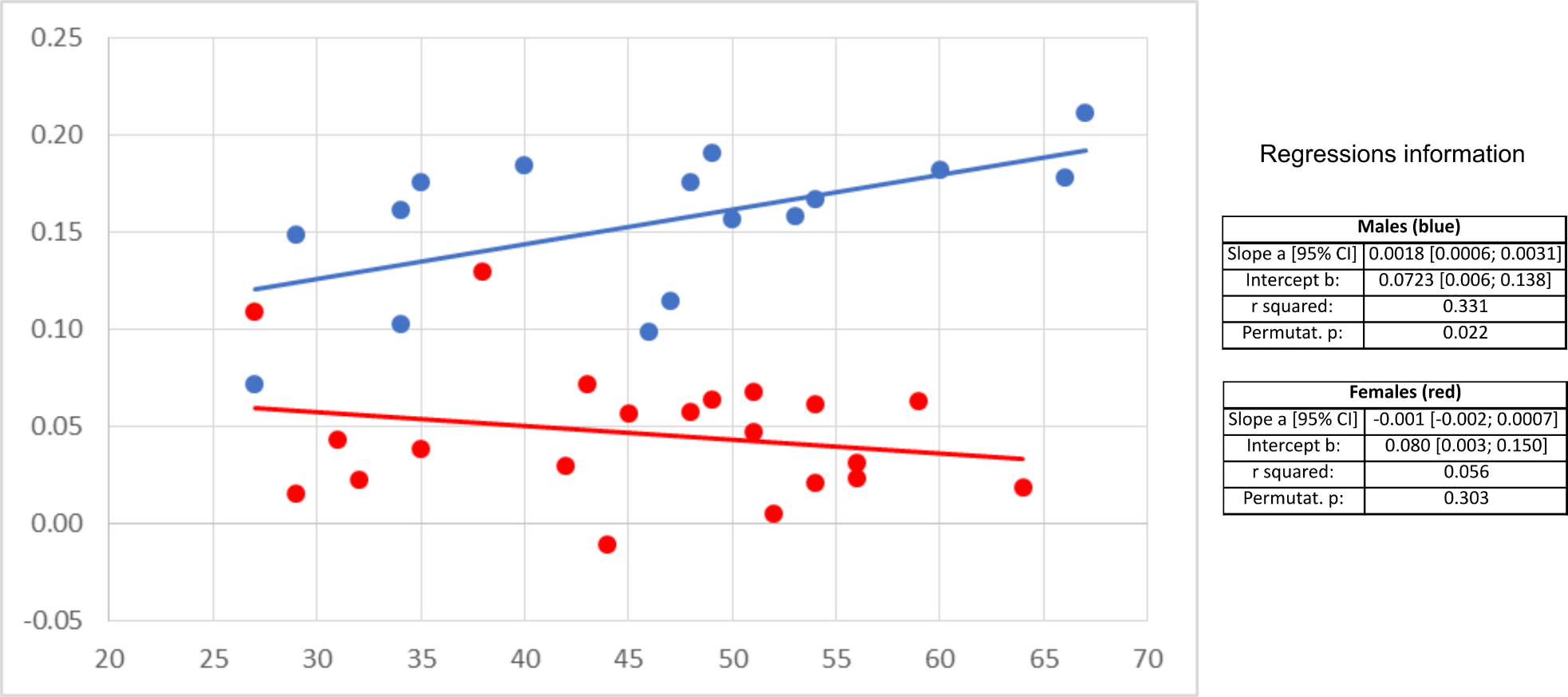


**Figure S7:** Second part of human thorax ontogeny. Depicted as linear regressions of shape scores (y-axis) on age (x-axis), showing males (blue) and females (red) adults (from 20 onwards). Regressions information is shown in the right part.

**Figure S8:** polynomial regression of stature (y-axis; mm) on T11 ventral height (x-axis; mm), males (blue) and females (red) showing parallel trajectories. These regressions are based on the combined data from the individuals of known stature from this paper (N=23) and the data from the provided by Dr. Marc Meyer (N=48). Those vertebrae derived from individuals 5–50 years of age at the time of death (n = 48; mean age = 23 years, median age = 22.5 years), with all juveniles having second molar eruption complete and at a stage of complete or near-complete vertebral development. Most of the modern human sample was from African Americans from the Hamann-Todd Osteological Collection housed at the Cleveland Museum of Natural History, USA. The sample also included indigenous San (n = 3) and Bantu individuals (n = 3) from the Ditsong National Museum of Natural History and the Dart collection, University of the Witwatersrand, South Africa and Kikuyu (n = 11) from the National Museums of Kenya.

**Table S1:** basic information from the individuals included in this study.

|  | **Origin** | **Sex** | **Development** | **CS** | **Age** | **T11 height** | **Stature** | **CS/Stature** |
| --- | --- | --- | --- | --- | --- | --- | --- | --- |
| eTX001 | Copenhagen | Male | Juvenile | 2344.16 | 9 | 15.59 | 138 | 16.99 |
| eTX002 | Copenhagen | Male | Juvenile | 2266.53 | 7 | 12.15 | 130 | 17.43 |
| eTX003 | Copenhagen | Male | Juvenile | 2121.7 | 8 | 12.24 | 127 | 16.71 |
| eTX004 | Copenhagen | Male | Juvenile | 2198.6 | 8 | 13.19 | 134 | 16.41 |
| eTX005 | Copenhagen | Male | Juvenile | 2554.87 | 10 | 14.6 | 153 | 16.7 |
| eTX006 | Copenhagen | Male | Juvenile | 2665.29 | 11 | 13.29 | 144 | 18.51 |
| eTX007 | Copenhagen | Male | Juvenile | 2566.5 | 11 | 14.8 | 167 | 15.37 |
| eTX008 | Copenhagen | Male | Juvenile | 2466.82 | 11 | 14.03 | 145 | 17.01 |
| eTX009 | Copenhagen | Male | Juvenile | 3171.03 | 12 | 15.66 | 175 | 18.12 |
| eTX010 | Copenhagen | Male | Juvenile | 2798.93 | 12 | 17.72 | 170 | 16.46 |
| eTX011 | Innsbruck | Male | Juvenile | 2386.46 | 10 | 19.78 | 168.13* | 14.19 |
| eTX012 | Innsbruck | Male | Juvenile | 2341.77 | 8 | 11.85 | 125.51* | 18.66 |
| eTX013 | Paris | Male | Adolescent | 3213.15 | 19 | 19.81 | 168.21* | 19.1 |
| eTX014 | Paris | Male | Adolescent | 3056.98 | 17.1 | 20.42 | 170.16* | 17.97 |
| eTX015 | Paris | Male | Adolescent | 3257.93 | 17.2 | 22.65 | 175.71* | 18.54 |
| eTX016 | Paris | Male | Adolescent | 2925.88 | 15.1 | 20.31 | 169.84* | 17.23 |
| eTX017 | Paris | Male | Adolescent | 2982.4 | 15 | 19.07 | 165.58* | 18.01 |
| eTX018 | Paris | Male | Adolescent | 2462.82 | 13.1 | 14.82 | 145.19* | 16.96 |
| eTX019 | Paris | Male | Adolescent | 2551.54 | 13.1 | 15.55 | 149.33* | 17.09 |
| eTX020 | Israel | Male | Adolescent | 3239.51 | 18 | 20.78 | 171.22* | 18.92 |
| eTX021 | New York City | Male | Adult | 3101.45 | 34 | 20.89 | 171.55* | 18.08 |
| eTX022 | Israel | Male | Adult | 3361.87 | 35 | 20.91 | 171.60* | 19.59 |
| eTX023 | Israel | Male | Adult | 3283.49 | 29 | 19.42 | 166.86* | 19.68 |
| eTX024 | Madrid | Male | Adult | 3362.38 | 66 | 22.69 | 174 | 19.32 |
| eTX025 | Madrid | Male | Adult | 3335.82 | 54 | 22.57 | 180 | 18.53 |
| eTX026 | Madrid | Male | Adult | 3316 | 34 | 20.96 | 171.74* | 19.31 |
| eTX027 | Madrid | Male | Adult | 3382.16 | 48 | 23.25 | 177 | 19.11 |
| eTX028 | Madrid | Male | Adult | 3172.02 | 47 | 20.22 | 166 | 19.11 |
| eTX029 | Madrid | Male | Adult | 3268.48 | 50 | 17.47 | 172 | 19 |
| eTX030 | Madrid | Male | Adult | 3283.47 | 53 | 21.28 | 171 | 19.2 |
| eTX031 | Madrid | Male | Adult | 3363.1 | 40 | 20.69 | 177 | 19 |
| eTX032 | Madrid | Male | Adult | 3241.35 | 44 | 21.01 | 173 | 18.74 |
| eTX033 | Madrid | Male | Adult | 3388.16 | 49 | 22.18 | 182 | 18.62 |
| eTX034 | Madrid | Male | Adult | 3467.52 | 67 | 21.4 | 170 | 20.4 |
| eTX035 | Madrid | Male | Adult | 3116.87 | 46 | 18.67 | 167 | 18.66 |
| eTX036 | Madrid | Male | Adult | 3391.13 | 60 | 21.77 | 176 | 19.27 |
| eTX037 | Madrid | Male | Adult | 3032.7 | 27 | 19.07 | 167 | 18.16 |
| eTX038 | Copenhagen | Female | Juvenile | 2202.13 | 7 | 12.53 | 117 | 18.82 |
| eTX039 | Copenhagen | Female | Juvenile | 2108.32 | 8 | 13.37 | 121 | 17.42 |
| eTX040 | Copenhagen | Female | Juvenile | 2252.51 | 8 | 15.58 | 134 | 16.81 |
| eTX041 | Copenhagen | Female | Juvenile | 2258.5 | 8 | 13.58 | 134 | 16.85 |
| eTX042 | Copenhagen | Female | Juvenile | 2411.74 | 9 | 9.83 | 139 | 17.35 |
| eTX043 | Copenhagen | Female | Juvenile | 2320.17 | 9 | 15.73 | 140 | 16.57 |
| eTX044 | Copenhagen | Female | Juvenile | 2346.11 | 10 | 15.11 | 143 | 16.41 |
| eTX045 | Copenhagen | Female | Adolescent | 2590.25 | 11 | 21.3 | 162 | 15.99 |
| eTX046 | Copenhagen | Female | Adolescent | 2698.34 | 12 | 19.08 | 160 | 16.86 |
| eTX047 | Copenhagen | Female | Adolescent | 2752.91 | 12 | 19.97 | 165 | 16.68 |
| eTX048 | Copenhagen | Female | Adolescent | 2556.32 | 12 | 17.15 | 162 | 15.78 |
| eTX049 | Copenhagen | Female | Adolescent | 2641.06 | 12 | 21.62 | 153 | 17.26 |
| eTX050 | Innsbruck | Female | Adolescent | 2698.42 | 11 | 17.75 | 150.45* | 17.94 |
| eTX051 | Innsbruck | Female | Adolescent | 2831.81 | 14 | 20.69 | 159.11* | 17.8 |
| eTX052 | Israel | Female | Adolescent | 2717.7 | 15.7 | 16.4 | 144.89* | 18.76 |
| eTX053 | Paris | Female | Adolescent | 3065.68 | 19.2 | 20.01 | 157.53* | 19.46 |
| eTX054 | Paris | Female | Adolescent | 2964.68 | 17.4 | 17.84 | 150.81* | 19.66 |
| eTX055 | Paris | Female | Adolescent | 2975.92 | 17.3 | 21.15 | 160.02* | 18.6 |
| eTX056 | Paris | Female | Adolescent | 2581.75 | 15.1 | 19.62 | 156.51* | 16.5 |
| eTX057 | Paris | Female | Adolescent | 2806.72 | 15.1 | 19.04 | 154.83* | 18.13 |
| eTX058 | Paris | Female | Adolescent | 2688.65 | 13.2 | 20.19 | 157.97* | 17.02 |
| eTX059 | Paris | Female | Adolescent | 2608.34 | 13 | 20.3 | 158.23* | 16.48 |
| eTX060 | Innsbruck | Female | Adult | 2907.66 | 35 | 22.53 | 162.05* | 17.94 |
| eTX061 | Innsbruck | Female | Adult | 2827.73 | 29 | 17.48 | 149.41* | 18.93 |
| eTX062 | Innsbruck | Female | Adult | 3097.06 | 27 | 22.42 | 161.93* | 19.13 |
| eTX063 | Madrid | Female | Adult | 2993.38 | 59 | 21.37 | 159 | 18.83 |
| eTX064 | Madrid | Female | Adult | 2981.07 | 54 | 18.46 | 155 | 19.23 |
| eTX065 | Madrid | Female | Adult | 2963.18 | 45 | 18.61 | 161 | 18.4 |
| eTX066 | Madrid | Female | Adult | 2822.14 | 52 | 21.42 | 158 | 17.86 |
| eTX067 | Madrid | Female | Adult | 3017.13 | 43 | 18.95 | 155 | 19.47 |
| eTX068 | Madrid | Female | Adult | 2815.72 | 44 | 22.47 | 162 | 17.38 |
| eTX069 | Madrid | Female | Adult | 2885.06 | 54 | 19.31 | 170 | 16.97 |
| eTX070 | Madrid | Female | Adult | 3000.04 | 49 | 20.19 | 165 | 18.18 |
| eTX071 | Madrid | Female | Adult | 2858.77 | 64 | 20.19 | 148 | 19.32 |
| eTX072 | Madrid | Female | Adult | 2960.44 | 51 | 17.56 | 149.73* | 19.77 |
| eTX073 | Madrid | Female | Adult | 2914.77 | 42 | 20.82 | 156 | 18.68 |
| eTX074 | Madrid | Female | Adult | 2913.17 | 56 | 24.4 | 163 | 17.87 |
| eTX075 | Madrid | Female | Adult | 2991.39 | 48 | 20.5 | 163 | 18.35 |
| eTX076 | Madrid | Female | Adult | 2894.67 | 56 | 21.07 | 161 | 17.98 |
| eTX077 | Madrid | Female | Adult | 3035.33 | 51 | 20.61 | 164 | 18.51 |
| eTX078 | Madrid | Female | Adult | 2972.85 | 31 | 17.63 | 166 | 17.91 |
| eTX079 | Paris | Female | Adult | 3197.79 | 38 | 20.89 | 159.51* | 20.05 |
| eTX080 | Paris | Female | Adult | 2876.97 | 32 | 19.38 | 155.84* | 18.46 |

* These values were estimated using the regressions from Fig. S8.
